# Polarization-controlled anisotropy in hybrid plasmonic nanoparticles

**DOI:** 10.1515/nanoph-2021-0691

**Published:** 2022-01-27

**Authors:** Xujie Wang, Zhenlong Dou, Chi Zhang, FangFang Deng, XiaoLin Lu, ShuangShuang Wang, Li Zhou, Tao Ding

**Affiliations:** Key Laboratory of Artificial Micro/Nano Structure of Ministry of Education, School of Physics and Technology, Wuhan University, Wuhan, 430072, China

**Keywords:** nanoparticles, nanorods, plasmons, polarization, two-photon, photolithography

## Abstract

Anisotropy has played a critical role in many material systems, but its controllable creation and modulation have been a long-lasting challenge for the scientific communities. Polarization-addressed anisotropy appears more attractive among all approaches due to its excellent controllability, simplicity, and accuracy, but only a limited number of material systems are applicable for such a concept, which are largely focused on oriented growth. Here, we establish a polarization-dependent anisotropic etching system made of Au@oligomer core–shell nanoparticles (NPs). As the oligomer coatings can be photochemically degraded via two-photon photolithography, the plasmonic near-field enhancement supported by the Au NP cores renders much faster degradation of the oligomer shells along the polarization, resulting in anisotropic Au@oligomer hybrid NPs. Such shape anisotropy leads to polarization-dependent photoluminescence with embedded dyes of methylene blue, which can be used as single-particle-based polarization detector. The oligomer lobes capped at the sides of the Au NP can also function as a protection agent for anisotropic photochemical growth of Au NPs, which evolve into Au nanorods and mushrooms with controlled irradiation time. Such polarization-directed etching of oligomer shells has unique advantages of high local-selectivity, controllability, and versatility for on-chip nanofabrication, which opens many new opportunities for integrated nanophotonic devices.

## 1 Introduction

Anisotropic nanoparticles (NPs) have attracted great research interests due to their pivotal role in material performances, which can potentially lead to compelling electric, photonic, mechanical, and biological functionalities [1]. Conventional synthetic approach via wet chemistry largely relies on facet control or ligand functionalization, which kinetically forms anisotropic NPs [2, 3]. Although this chemical approach is capable of yielding a batch of NPs in a scalable manner, the uniformity and the reproducibility are not always robust, and such a batch of dispersion does not present any anisotropic properties as an entity due to the randomness of the NPs therein. Thus, additional assembly or post-processing steps are required to align them in the same orientation for the desired functionality [4], [5], [6]. Physical methods like physical vapor deposition [7], e-beam lithography [8], nanoimprint lithography [9], and glancing angle deposition [10] can reliably produce individual nanocomponents demonstrating prominent anisotropic properties, but the complication and the cost-effectiveness of fabrication render any of their application commercially difficult.

Anisotropy created via the polarization of light is much facile and delicate as they can be easily tailored by polarization optics at ambient conditions [11, 12]. Although several interesting demonstration of polarization-induced material modification can lead to oriented nanostructures, most of them are beyond the diffraction limit of light with sizes over a few microns [13], [14], [15], [16]. Plasmon-assisted near-field lithography due to the electric dipole induced by the linearly polarized incidence has shown strong capability of fabricating nanomaterials much below the diffraction limit, which can be used for near-field mapping [17], [18], [19], 5D data storage [20], and photoelectric nanoswitches [21]. However, most of the prior arts are based on polarization-directed polymerization with high material constraint, and little functionality relating to their anisotropic features were demonstrated [22], [23], [24], [25], [26], [27], [28], [29].

Here, instead of using near-field polarization to control the growth of polymers, we exploit plasmon-enhanced photochemical degradation of the oligomer shells around the Au NPs selectively along polarization, which eventually forms anisotropic coating at the opposite sides of the Au NPs. When the dye molecules are embedded within the oligomers, the etched Au@oligomer hybrid NPs present polarization-dependent photoluminescence (PL) property. The oligomer lobes at the sides of the Au NP can also function as a protecting agent on the Au surface, which renders one-dimensional growth of the Au nanorod (NR) with tailorable shapes. Such Au@oligomer hybrid particle system is tailorable with irradiation time, which evokes intriguing applications for single-particle-based polarization detector as well as particle caps for anisotropic engineering of Au NP shapes and their plasmonic properties.

## 2 Experimental Methods

### 2.1 Synthesis of Au@oligomer core–shell NPs

About 1 mL of 80 nm Au NPs (from Nanopartz) was mixed with 200 μL aqueous solution of 2-hydroxy-2-methylpropiophenone (HMPP) (0.1 v%), followed by UV irradiation (302 nm) for 15 min. The Au@oligomer core–shell NPs were purified by centrifugation, which were finally stored in deionized (DI) water for further use. For methylene blue (MB)-doped Au@oligomer core–shell NPs, 0.5 mM of MB was added in the mixture before UV irradiation, which embedded the MB molecules in the oligomer layer.

### 2.2 Polarization-directed selective etching of the oligomer shells

The Au@oligomer NPs were drop-casted on Au films with a thickness of 100 nm or Si substrate and rinsed with DI water. The samples were irradiated with continuous wave (CW) lasers of different wavelengths (641 nm, 532 nm, 446 nm) using a dark field (DF) microscope (Olympus BX35). The lasers were focused on the samples through a ×100 DF objective (Olympus, NA = 0.8), and the scattering spectra were collected confocally through a 50 μm fiber coupled to an optofiber spectrometer (QEPro, Ocean Optics). The polarization of the lasers was attenuated with a linear polarizer and a half-wave plate. Irradiation power and time were varied to investigate their influence on the morphology of the final products.

### 2.3 Protected overgrowth of Au NRs

The anisotropic Au@oligomer NPs were immersed in HAuCl_4_ aqueous solution (3 mM) and placed under a microscope with a coverslip for a laser-induced growth of Au NPs. A 641 nm CW laser was selected as the excitation beam to avoid any possible photochemistry. The irradiation power was set at 1.5 mW with a duration varying from 0 to 20 s. After finishing the irradiation, all samples were rinsed with DI water to remove excess HAuCl4, which were blow-dried with a nitrogen gun for scattering spectroscopy measurements and SEM characterizations.

### 2.4 Characterizations

UV–vis extinction spectra of the Au NPs and hybrid core–shell NPs were recorded with an optofiber spectrometer (QE65000, Ocean Optics). The Z-scan measurement of the oligomer solution (60 mM) was performed with a femtosecond Ti-sapphire laser (Mira900, Coherent) at a wavelength of 760 nm (3 ps pulse width with a repetition rate of 76 MHz). SEM images of the Au@oligomer NPs before and after irradiation were captured at an accelerating voltage of 10 kV (Sigma Zeiss). Polarization-dependent PL spectra of the MB-doped Au@oligomer were taken using a pulsed laser beam at 633 nm (10 μW).

### 2.5 Simulations

Finite difference time domain (FDTD) method was employed to calculate the electric-field profile around the Au@oligomer NPs during laser (641 nm) irradiation. The finest mesh size is set at 0.5 nm. The refractive index (RI) of Au is adopted from Johnson and Christy, and the refractive index (RI) of the oligomer is set at 1.527.

## 3 Results and discussion

**Figure 1:**
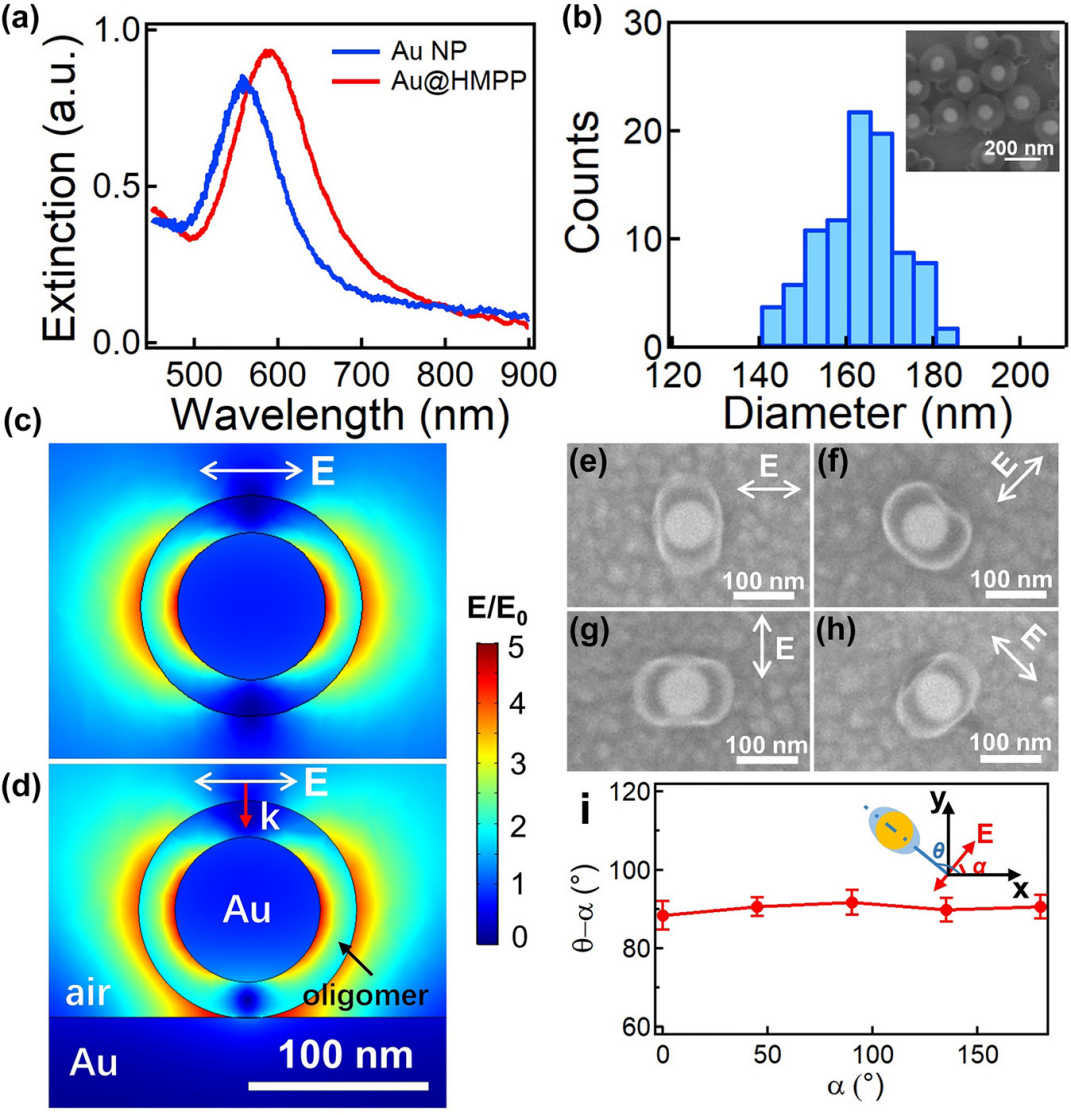
Polarization-directed etching of Au@oligomer hybrid NPs. (a) UV–vis extinction spectra of Au and Au@oligomer core–shell NPs. (b) Size distribution of the as-synthesized Au@oligomer core–shell NPs. Inset is their typical SEM image. (c) and (d) Simulated electric near-field profile of the Au@oligomer core–shell NPs on Au films: (c) top view, (d) cross-sectional view. The incidence is a linearly polarized plane wave with a wavelength of 641 nm. (e)–(h) SEM images of the etched Au@oligomer hybrid NPs with different directions of polarization. The irradiation power was 0.07 mW. (i) Correlation of the long axial direction (*θ*) of the etched Au@oligomer hybrid NPs and the polarization direction (*α*) of the irradiation beam. Inset is the scheme defining the angles of long axial direction of the etched Au@oligomer hybrid NPs and the polarization direction.

The Au@oligomer core–shell NPs were synthesized via photoinduced self-initiation of HMPP, as reported previously [30]. The photochemical cleavage of *α*-C in HMPP via Norrish type I reaction generates free radicals which can self-initiate to form oligomers of ∼5–10 repeating units. Such oligomers have low solubility in water, which nucleate and grow on the surface of Au NPs to form Au@oligomer core–shell NPs. The UV–vis spectra of Au NPs redshift from 559 to 590 nm (Figure 1(a)), suggesting successful coating of the oligomer due to the RI increase in the surrounding medium. SEM characterization further verifies the uniform core–shell feature of the Au@oligomer NPs (inset of Figure 1(b)) with a shell thickness of 40 ± 10 nm (Figure 1(b)). Because of the low molecular weight of such HMPP oligomers, they can be photochemically degraded with irradiation of CW laser via two-photon dissociation (Supplementary materials (SM) Figure S1) [31], [32], [33]. The two-photon absorption coefficient ($\beta_{eff}$) of the oligomers is 1.5 cm/GW with an absorption cross-section (*δ*) of ∼10^3^ GM. Electric near-field simulation via FDTD method reveals strong local *E* field along the polarization of the laser beam (Figure 1(c) and (d)), which enhances the local photochemical degradation kinetics of oligomers along the direction of polarization [26, 34, 35], leading to anisotropic etching of the oligomer shells. As a result, we observe that the etching direction (short axis of the Au@oligomer NPs) changes with the polarization direction (Figure 1(e)–(h)), whereas the long axes are mostly perpendicular to the polarization direction with an angle of 90 ± 1° (Figure 1(i)). Such polarization-dependent etching also rules out the possibility of photothermal-induced degradation of the oligomer shells as it normally leads to uniform degradation. Other polymer coatings such as poly(divinylbenzene) (PDVB) can also be degraded along the polarization, forming Janus-like Au/PDVB hybrid NPs (SM, Figure S2).

**Figure 2:**
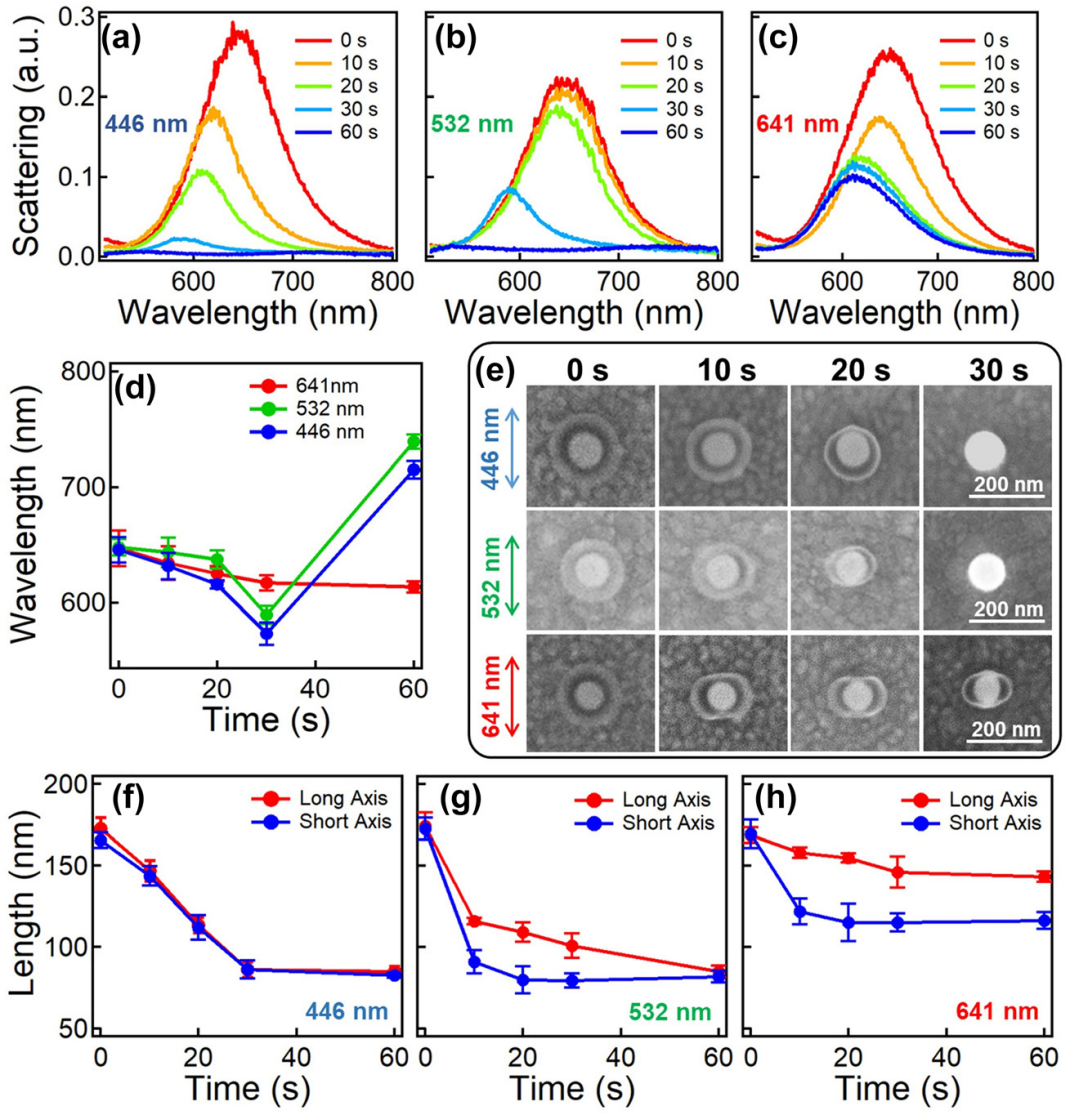
Wavelength-dependent etching of Au@oligomer hybrid NPs. (a)–(c) Scattering spectra kinetics of Au@oligomer hybrid NPs irradiated with (a) 446 nm, (b) 532 nm, and (c) 641 nm lasers. The irradiation power was all set at 0.07 mW. (d) Change of plasmon resonances with irradiation time. The data are extracted from (a)–(c) correspondingly. (e) SEM images of the Au@oligomer hybrid NPs under the irradiation of linearly polarized lasers of 446, 532, and 641 nm at time points of 0, 10, 20, and 30 s. The arrows indicate the polarization direction of the laser beam. (f) and (g) are the corresponding size changes of Au@oligomer hybrid NPs at the irradiation wavelength of (f) 446 nm, (g) 532 nm, and (h) 641 nm.

The degradation rate of HMPP oligomer is highly dependent on the photon energy, thus the etching kinetics of the HMPP oligomers is different for irradiation with different wavelength lasers (Figure 2(a)–(c)). In all the cases, the DF scattering spectra blueshift with irradiation time, which is due to the reduced RI of the surrounding medium induced by the near-field etching of HMPP oligomers. The oligomer chain length also affects the efficiency of two-photon dissociation, where the longer the chains are, the less it degrades along the polarization, which results in reduced aspect ratios (SM, Figure S3). But, for 446 and 532 nm light, the HMPP oligomers can be photochemically degraded directly via single-photon excitation, which leads to uniform etching of the oligomer beads at lower power even without the presence of Au NPs (SM, Figure S1(e) and (f)); thus, it presents much faster degradation kinetics with dramatic attenuation of the plasmon resonances, which completely etches the shells after 30 s (Figure 2(e)). Since the gap between the Au NP and Au films decreases, the plasmon resonances show redshift after 30 s irradiation (blue and green lines in Figure 2(d) and SM, Figure S4). The uniform etching via single-photon dissociation counteracts the polarization-induced anisotropic etching, which eventually leads to no anisotropy in the final plasmonic nanostructures. Therefore, we only observe anisotropic etching of HMPP oligomers with 641 nm laser irradiation. The size-change kinetics also reveals how such anisotropy generates (Figure 2(f)–(h)). The size of the HMPP oligomer coating decreases uniformly with 446 nm laser irradiation (Figure 2(f)) but non-uniformly with the irradiation of 641 nm laser (Figure 2(h)). With 532 nm laser, the Au@oligomer core–shell NP becomes anisotropic at an early stage of irradiation, which diminishes as the HMPP oligomer shells are completely etched (Figure 2(g)).

**Figure 3:**
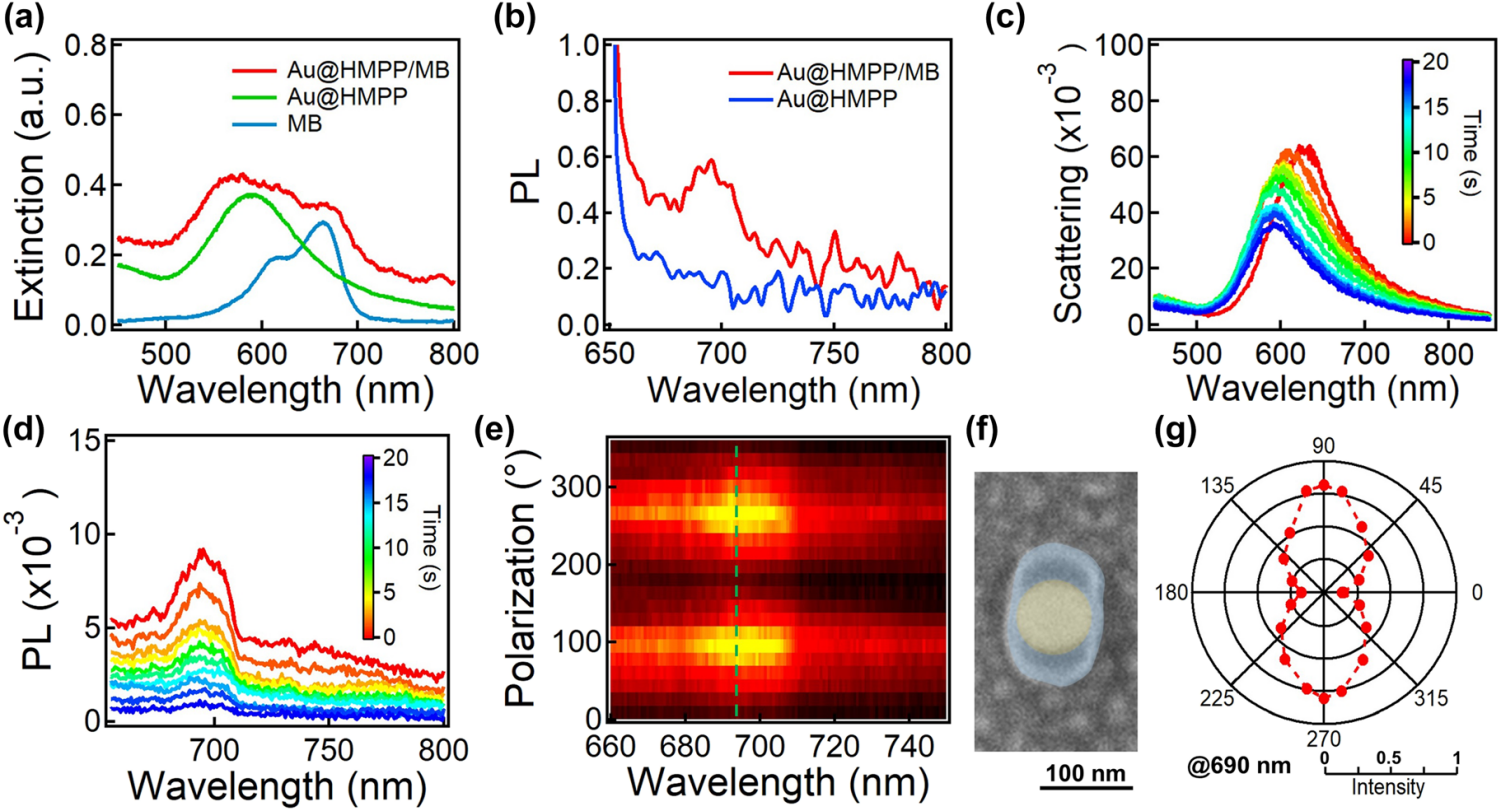
Polarization-dependent PL properties of Au@oligomer hybrid NPs. (a) UV–vis extinction spectra of Au@oligomer/MB, Au@oligomer, and Au NPs. (b) PL spectra of Au@oligomer/MB and Au@oligomer NP on Au films. Change of (c) scattering and (d) PL spectra with irradiation time. Irradiation power is 0.01 mW. (e) Polarization-dependent PL spectra of an etched Au@oligomer hybrid NP. The green dashed line marks the PL peak at ∼590 nm. (f) SEM image of the corresponding etched Au@oligomer hybrid NP. The fake-colored particle highlights the composition differences. (g) Polar plot of the PL intensity (at 690 nm) with the polarization angle of the excitation laser. The data are extracted from (e).

As plasmon-enhanced PL is highly related to the alignment of the electric field direction and molecular dipole, polarization-directed anisotropic etching also controls the spatial distribution of the dye molecules in an asymmetric manner, which results in polarization-dependent excitation of single plasmonic hybrid NP. Here, we embedded the dye molecules of MB in the HMPP oligomer coating layer during the photoinitiation process (see Experimental Methods section for details). The UV–vis spectrum of the MB-doped Au@oligomer NPs shows extinction peak of both plasmons and dyes (Figure 3(a)), suggesting a successful coating of HMPP oligomers along with the MB molecules. Clearly, the MB-doped Au@oligomers show a PL peak at 690 nm, whereas pure Au@oligomer NPs show no PL peak (Figure 3(b)). Irradiation of the Au@oligomers with a 641 nm CW laser etches the HMPP oligomer shells selectively along polarization, thus the scattering spectra of the Au@oligomer/MB gradually blueshifts due to the decrease of RI (Figure 3(c)). Meanwhile, the PL intensity drops consecutively with irradiation time due to the photodegradation of the oligomer/MB shells along polarization (Figure 3(d)). Such a decrease in intensity suggests photodegradation of MB along with the oligomer, as evidenced by the kinetic Raman spectra (SM, Figure S5). However, the MB molecules in the perpendicular direction of the polarization remain almost intact, which show a prominent PL if excitation polarization is aligned along the long axis of Au@oligomer hybrid NP (Figure 3(e)). As such, the PL property of such anisotropic Au@oligomer/MB shows strong polarization-dependent excitation with the strongest intensity excited along the long axis of the Au@oligomer hybrid NP (Figure 3(f) and (g)), whereas the PL intensity of uniform core–shell NPs is found to be constant with the excitation of different polarizations (SM, Figure S6). Such an anisotropic colloidal emitter can be easily integrated in nanophotonic devices as a polarization optical detector.

**Figure 4:**
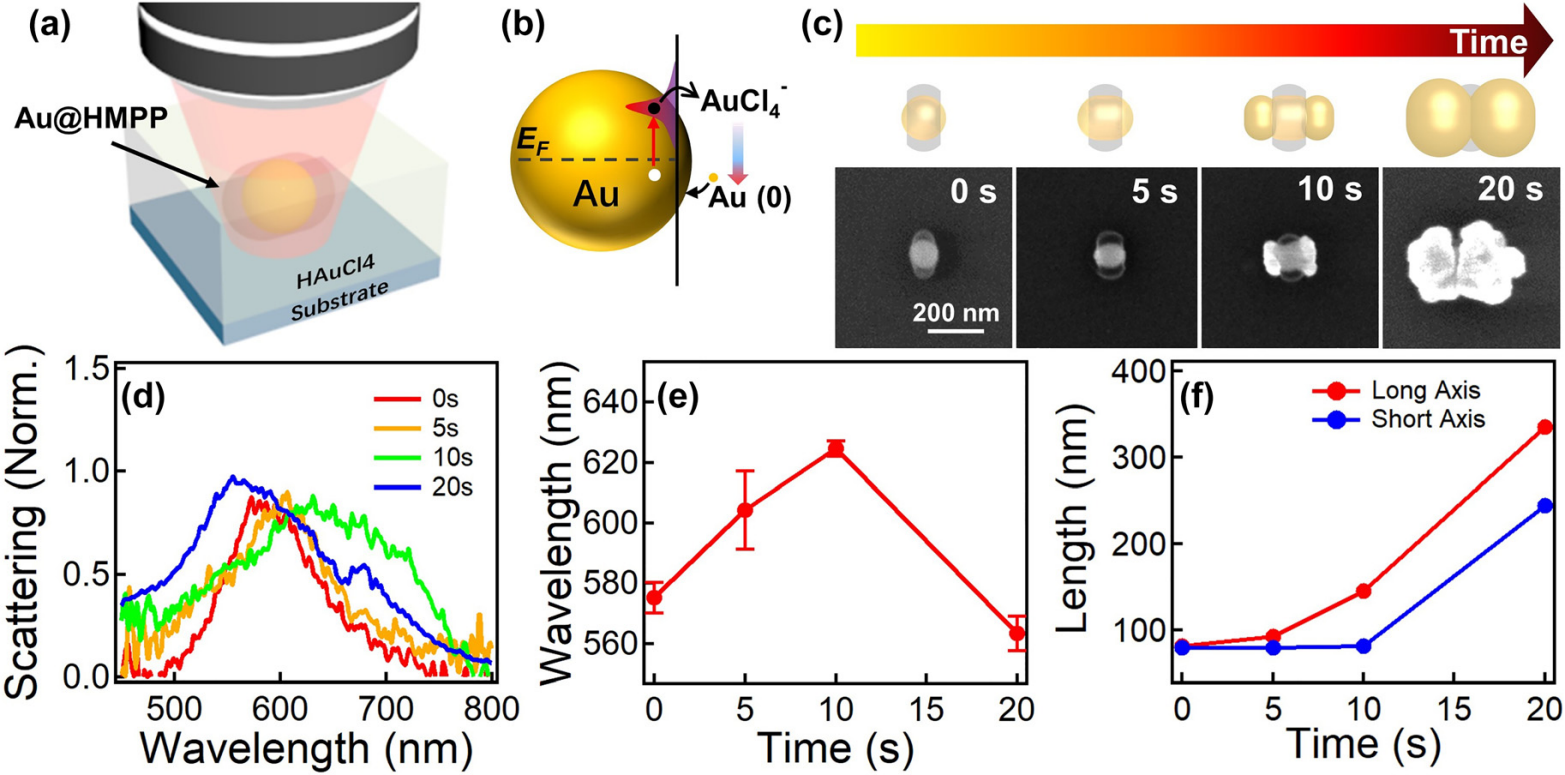
Oligomer-capped anisotropic growth of Au NRs. Schemes of experimental setup for plasmon-mediated optical growth of Au NPs and (b) mechanism. (c) Growth of the Au NRs with irradiation time. Top panel includes the schemes of the overgrown Au/oligomer hybrid NPs and bottom panel includes the corresponding SEM images. (d) Normalized scattering spectra of the Au/oligomer hybrid NPs at different time of irradiation and (e) the change of plasmon resonances with time. The laser wavelength is 641 nm, and the irradiation power is 1.5 mW. (f) Change of the length of Au NR with irradiation time.

The oligomer lobes capped at the side of the Au NPs can also function as a physical capping agent in analogue to Cetrimonium Bromide (CTAB) for anisotropic growth of Au NRs, and here, it is more robust and stable for on-chip growth, which is beneficial for *in situ* fabrication and integration [36]. We use a 641 nm CW laser to further trigger the anisotropic growth of Au NPs under the protection of the oligomer lobes at the sides (Figure 4(a)), which is monitored with DF scattering spectroscopy. The growth is driven by a hot-electron-induced reduction of HAuCl_4_, which forms Au (0) for epitaxial growth of Au NPs, as verified previously (Figure 4(b)) [37]. Such a growth selectively happens at the location without any physical protection of oligomers, forming bone-like Au NRs. Further growth with prolonged irradiation results in mushroom-shaped Au caps, which back-encapsulate the oligomers to minimize the surface-to-volume ratio. This is reasonable as the shielding effect offered by the HMPP oligomers fails at the ends that are far from the oligomer lobes (Figure 4(c)). As a result, the plasmon resonances change dramatically, which can be controlled with irradiation time (Figure 4(d) and (e)). The redshift is due to the increasing aspect ratio of Au NR, whereas the abrupt blueshift is caused by the contact of the Au mushroom caps during the back-encapsulation. The length of the Au NPs increases anisotropically at an early stage of irradiation but then (>10 s) uniform growth prevails, which leads to almost equal increase in both long and short axes (Figure 4(f)). Such peculiar shape evolution model of Au NPs is not common in traditional ligand-protected growth as the latter is always abundant. Here, the limited protection length of the oligomer lobes at the side of the Au NPs enables diverse engineering of the particle shapes simply by changing the irradiation time.

## 4 Conclusions

We have shown a hybrid plasmonic NP system made of Au@oligomer that can be anisotropically etched via linearly polarized CW laser. The etching direction appears the same as the direction of the polarization, making their orientation facilely controllable and tunable. Such a polarization-dependent etching is related to the near-field enhanced two-photon photolithography which selectively degrades the oligomer coating in the direction of polarization. Dye molecules embedded in such an anisotropic hybrid plasmonic NP system presents polarization-dependent excitation which can be used as a polarization nanodetector. Moreover, the oligomer lobes also confine the optical overgrowth of Au NP in one dimension, which forms Au NRs, bone-shaped Au NRs, and mushroom-shaped Au NPs, depending on the irradiation time. Such polarization-directed anisotropic etching takes place on single NP level, which not only enriches the polarization-directed nanofabrication but also brings many new opportunities for on-chip integration and *in situ* monitoring of nanophotonic devices.

## Supplementary Material

Supplementary Material
