# Supplementary material for: Polarization-controlled anisotropy in hybrid plasmonic nanoparticles
Source: Nanophotonics. 2022 Jan 27;11(5):1003–9. doi: 10.1515/nanoph-2021-0691 (PMC11501720; doi:10.1515/nanoph-2021-0691)
Supplement: Supplementary file 1 — Supplementary Material [file j_nanoph-2021-0691_suppl.docx]

Supplementary Materials

Polarization-Controlled Anisotropy in Hybrid Plasmonic Nanoparticles

Xujie Wang, Zhenlong Dou,Chi Zhang, FangFang Deng, XiaoLin Lu, ShuangShuang Wang, Li Zhou and Tao Ding*

Key Laboratory of Artificial Micro/Nano Structure of Ministry of Education, School of Physics and Technology, Wuhan University, Wuhan, 430072, China.

*Email: t.ding@whu.edu.cn

Two-photon photodegradation mechanism:

The oligomer beads only present a strong absorption in the UV (~350 nm) in the extinction spectra whereas no clear absorption was found in the wavelength above 600 nm (Figure S1a). Thus, it is likely such photodegradation proceeds via two-photon absorption mechanism (Figure S1b, c). The two-photon absorption coefficient of the oligomers can be determined via Z-scan measurement (Figure S1d), which suggests a nonlinear coefficient of $\beta_{eff}$=1.5 cm/GW and absorption cross-section of 10^3^ GM. The beads of HMPP oligomers can be photochemically degraded with 641 nm laser irradiation at high power (Figure S1g) and for the irradiation with shorter wavelength, the degradation is much stronger (Figure S1e, f) likely also involve some single photon absorption process.


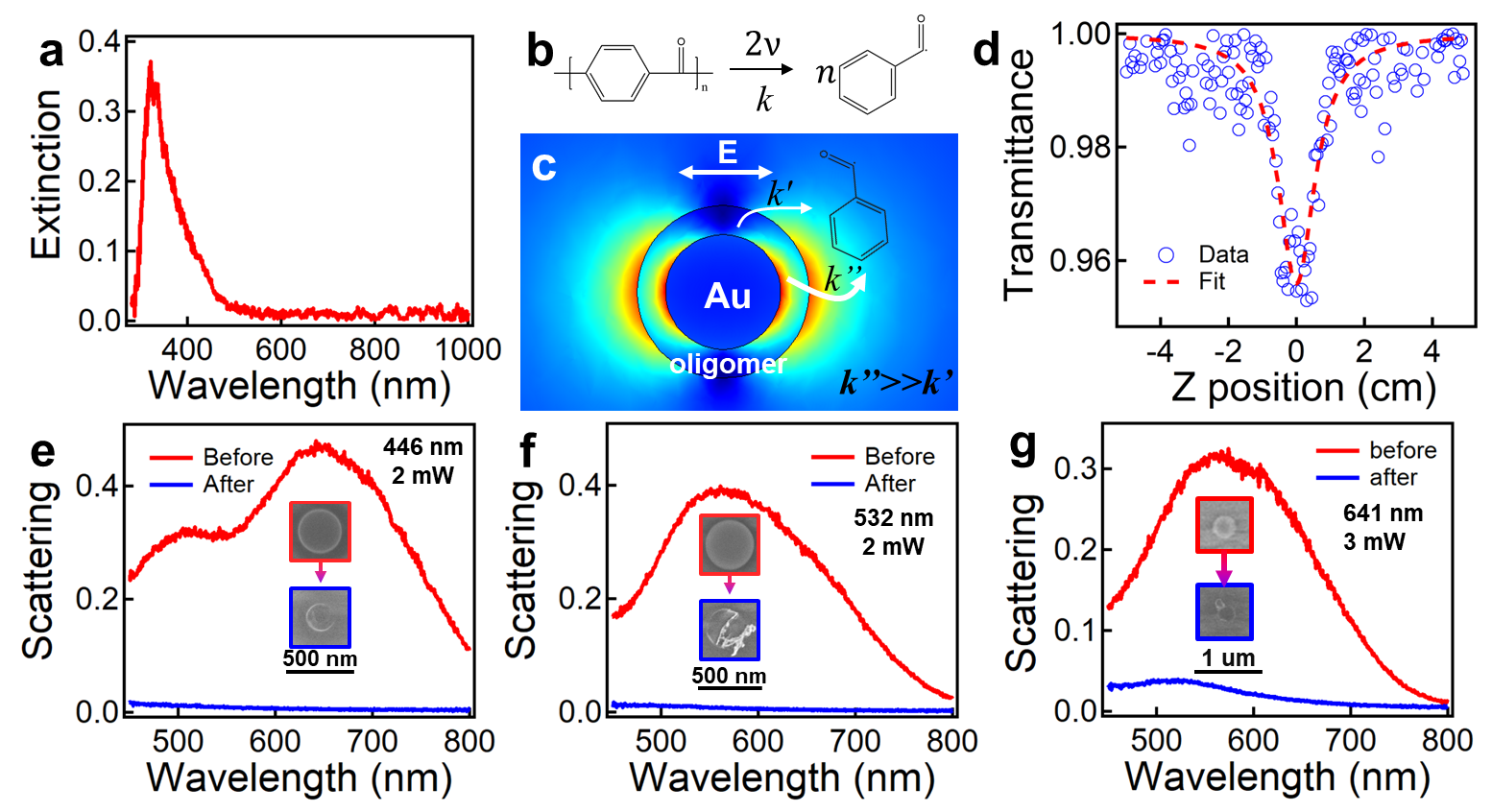


**Figure S1: Mechanism of plasmon enhanced two-photon degradation.** (a) UV-Vis spectra of pure HMPP oligomers dispersed in water. (b) Molecular scheme of two-photon disassociation and (c) near field profile of Au@oligomer under linear polarization, indicating boosted photodegradation kinetics along the polarization. (d) Z-scan spectra of HMPP oligomer solution. (e-g) Scattering spectra of pure HMPP oligomer particles before and after irradiation. The irradiation condition is (e) 2 mW, 446 nm laser, (f) 2 mW, 532 nm laser, (g) 3 mW, 641 nm laser. Insets are the corresponding SEM images of the particles before and after irradiation.


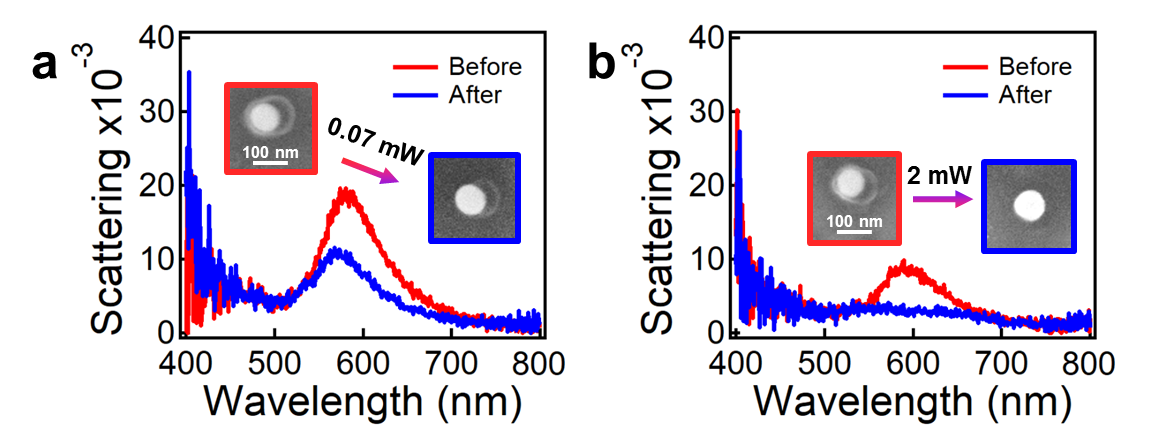


**Figure S2: Laser irradiation of Au@Poly(divinylbenzene) NP**. Scattering spectra before and after irradiation with polarization parallel to the long axis. The irradiation power is (a) 0.07 mW, (b) 2 mW.


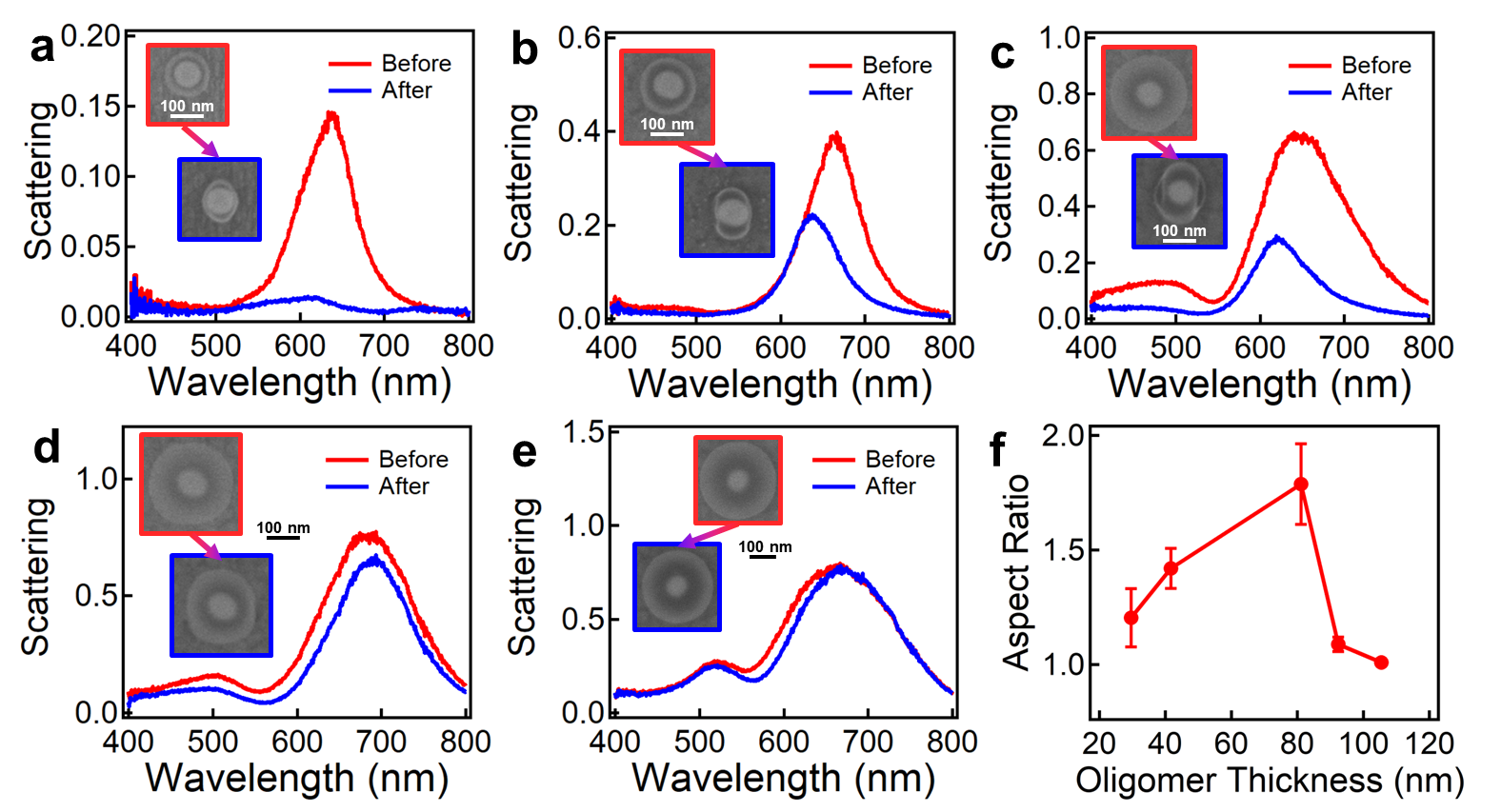


**Figure S3: Influence of oligomer chain length on the photodegradation efficiency.** (a-e) Scattering spectra of the Au@oligomer before and after laser irradiation (641 nm, 0.1 mW). Insets are the corresponding SEM images before and after irradiation. (f) Change of aspect ratio with the thickness of oligomer coating. Note the oligomer chain length is proportional to the thickness as more HMPP is added for higher degree of polymerization.


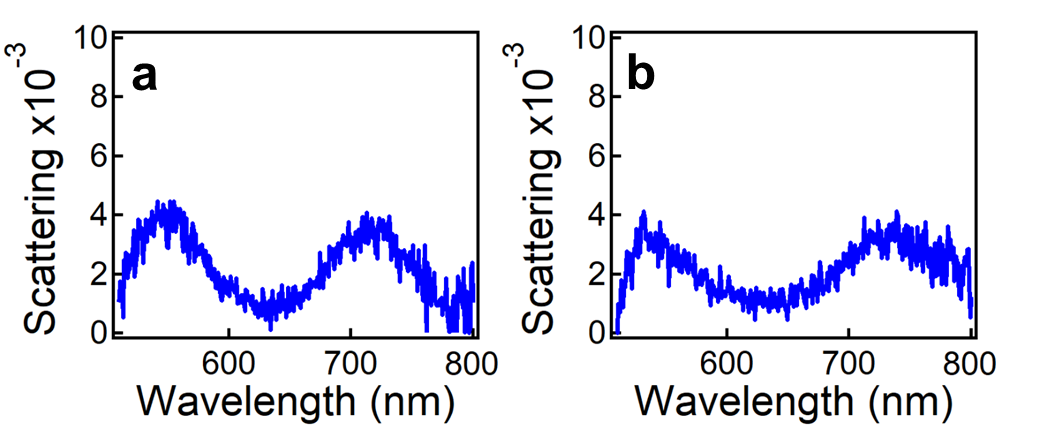


**Figure S4: Scattering spectra of Au@oligomer NPoM after 60s’ irradiation.** (a) 446 nm laser, 0.07 mW, (b) 532 nm laser, 0.07 mW.


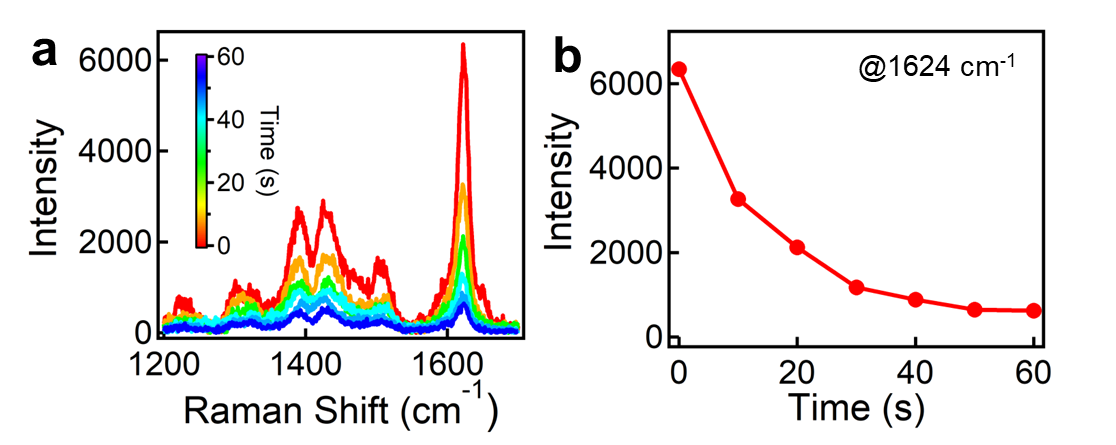


**Figure S5: Raman analysis of MB during the etching process.** (a) Change of Raman spectra with irradiation time. Laser wavelength：633 nm; Power: 0.05 mW. (b) Change of Raman peak intensity (1624 cm^-1^) with time.

For uniform Au@oligomer core-shell NPs, we change the excitation polarization and detect the PL signal, from which we found their intensity is almost the same irrespective of the polarization directions (Figure S6).


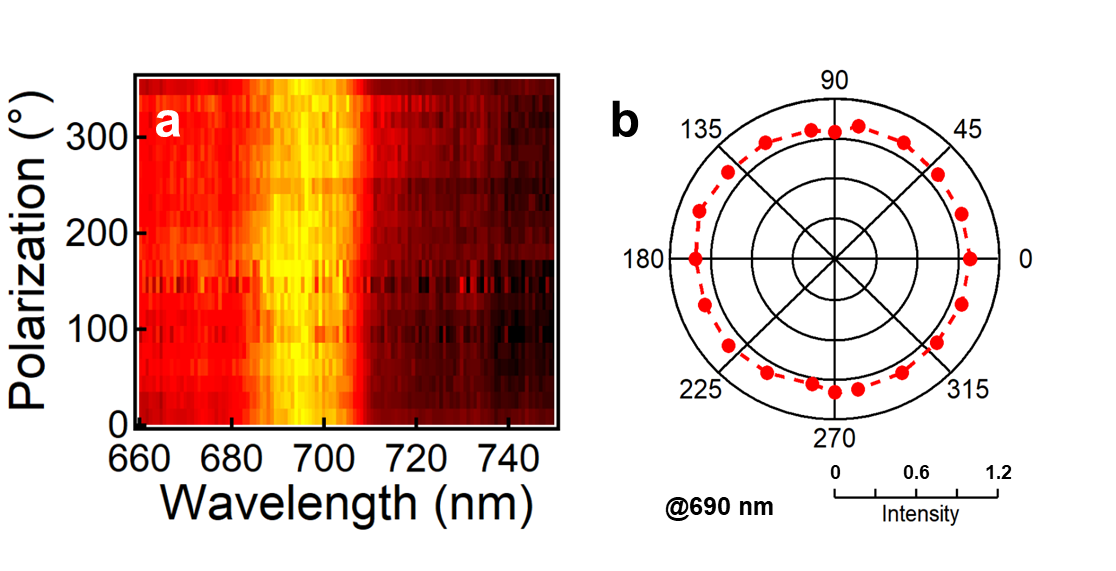


**Figure S6: PL spectra of Au@oligomer/MB core shell NPs.** (a) PL spectra of Au@oligomer/MB core-shell NP excited with different polarizations (633 nm) and (b) its intensity change with the polarization direction.
